# Suppressive Effects of 4-(Phenylsulfanyl) Butan-2-One on CCL-1 Production via Histone Acetylation in Monocytes

**DOI:** 10.3390/cimb44100315

**Published:** 2022-10-03

**Authors:** Ming-Kai Tsai, Mei-Lan Tsai, Zhi-Hong Wen, Wei-Ting Liao, Yi-Ching Lin, Hsin-Ying Clair Chiou, Ming-Hong Lin, Chih-Hsing Hung

**Affiliations:** 1 Division of Nephrology, Department of internal Medicine, Kaohsiung Armed Forces General Hospital, Kaohsiung 802, Taiwan; 2 Department of Marine Biotechnology and Resources, National Sun Yat-sen University, Kaohsiung 804, Taiwan; 3 Institute of Medical Science and Technology, National Sun Yat-sen University, Kaohsiung 804, Taiwan; 4 Graduate Institute of Medicine, College of Medicine, Kaohsiung Medical University, Kaohsiung 807, Taiwan; 5 Department of Pediatrics, Faculty of Pediatrics, College of Medicine, Kaohsiung Medical University, Kaohsiung 807, Taiwan; 6 Department of Marine Biotechnology, National Sun Yat-sen University, Kaohsiung 804, Taiwan; 7 Institute of Biopharmaceutical Sciences, National Sun Yat-sen University, Kaohsiung 804, Taiwan; 8 Department of Medical Research, Kaohsiung Medical University Hospital, Kaohsiung Medical University, Kaohsiung 807, Taiwan; 9 Department of Biotechnology, College of Life Science, Kaohsiung Medical University, Kaohsiung 807, Taiwan; 10 Research Center for Environmental Medicine, Kaohsiung Medical University, Kaohsiung 807, Taiwan; 11 Department of Laboratory Medicine, Kaohsiung Medical University Hospital, Kaohsiung Medical University, Kaohsiung 807, Taiwan; 12 Doctoral Degree Program of Toxicology, College of Pharmacy, Kaohsiung Medical University, Kaohsiung 807, Taiwan; 13 Department of Laboratory Medicine, School of Medicine, College of Medicine, Kaohsiung Medical University, Kaohsiung 807, Taiwan; 14 Teaching and Research Center of Kaohsiung Municipal Siaogang Hospital, Kaohsiung 812, Taiwan; 15 Department of Microbiology and Immunology, School of Medicine, College of Medicine, Kaohsiung Medical University, Kaohsiung 807, Taiwan; 16 Department of Pediatrics, Kaohsiung Medical University Hospital, Kaohsiung Medical University, Kaohsiung 807, Taiwan; 17 Department of Pediatrics, Kaohsiung Municipal Siaogang Hospital, Kaohsiung 812, Taiwan

**Keywords:** 4-(phenylsulfanyl)butan-2-one, CCL-1, monocyte, histone acetylation

## Abstract

The 4-(phenylsulfanyl) butan-2-one (4-PSB-2), a marine-derived compound from soft coral, was proven to have multiple biological activities including neuroprotection and potent anti-inflammatory effects. CC chemokine ligand (CCL)-1 belongs to T helper (Th)2-related chemokines that are involved in the recruitment of Th2 inflammatory cells. Histone acetylation has been recognized as a critical mechanism underlying the regulated cytokine and chemokine production. Our study tried to investigate the anti-inflammatory effect of 4-PSB-2 on CCL-1 production in human monocytes and explore possible underlying intracellular processes, including epigenetic regulation. To confirm our hypothesis, human monocyte THP-1 cell line and primary CD14^+^ cells were pretreated with various concentrations of 4-PSB-2 and then were stimulated with lipopolysaccharide (LPS). The CCL-1 concentration was measured by enzyme-linked immunosorbent assays, and the intracellular signaling pathways and epigenetic regulation of 4-PSB-2 were investigated by using Western blotting and chromatin immunoprecipitation analysis. In this study, we found that 4-PSB-2 had a suppressive effect on LPS-induced CCL-1 production. Moreover, this suppressive effect of 4-PSB-2 was mediated via intracellular signaling such as the mitogen-activated protein kinase and nuclear factor-κB pathways. In addition, 4-PSB-2 could suppress CCL-1 production by epigenetic regulation through downregulating histone H3 and H4 acetylation. In short, our study demonstrated that 4-PSB-2 may have a potential role in the treatment of allergic inflammation.

## 1. Introduction

Bio-functional compounds from natural products have been important in new drug development for a long time. Austrasulfone and dihydroaustrasulfone alcohol, two chemical compounds extracted from soft corals Cladiella australis, have been known to have multiple bio-functions including neuroprotection with anti-inflammation activity [1,2,3,4,5]. Austrasulfone was first recognized as a bioactive substance that expresses neuroprotective and anti-inflammatory effects by Wen et al. [6]. The 4-(phenylsulfanyl) butan-2-one (4-PSB-2) is a substance with an anti-inflammatory activity that was adapted from dihydroaustrasulfone alcohol, a synthetic precursor of austrasulfone [7]. In 4-PSB-2, a benzene ring was used to replace the polar hydroxyl group in hydroxylated sulfone to increase lipophilicity and binding affinity to the cell membrane [7,8]. The anti-inflammatory effects of 4-PSB-2 are exerted via suppressing lipopolysaccharide (LPS)-related inducible nitric oxide synthase (iNOS) in murine macrophages [5]. Moreover, the anti-melanogenic ability of 4-PSB-2 has been proven by suppressing tyrosinase activity in zebrafish [8]. Previous research also revealed that 4-PSB-2 has neuroprotective effects in amyloid-beta-induced toxicity in retinal pigment epithelium cells [9].

Inappropriate T helper (Th) 1/Th2 balance plays a major part in the pathogenesis of autoimmune and allergic diseases. Allergic diseases are cell-mediated immune responses characterized by the predominant infiltration of Th2 lymphocytes with inflammatory cells [10]. Inflammatory cells were recruited to the inflammation area by signal proteins called chemokines, and thus they have critical roles in the development of allergic diseases [11]. CC chemokine ligand (CCL)-1 is a Th2-related chemokine produced by multiple cells such as mononuclear cells including monocytes and endothelial cells. Previous research has revealed its ability in recruiting Th2 cells in response to allergen challenges [12].

Histone modification, one of epigenetic regulation, involves alterations in gene expression that occur without direct changes in the DNA sequence. Previous studies had revealed that the severity of bronchial hyperresponsiveness is associated with increased acetylation activity in children with asthma [13]. In CD4+ T cells from asthmatic children, the acetylation level of histone H3 and H4 were increased, and histone H3 acetylation was also significantly correlated with higher interleukin (IL)-13 concentration [14]. Therefore, histone modification was considered an important mechanism in the pathogenesis of allergy disorders [15].

As mentioned above, previous research has revealed 4-PSB-2 with anti-inflammation activity in animal experiments; however, the underlying pathways of these anti-inflammatory effects in 4-PSB-2 are rare. In this study, we hypothesized that 4-PSB-2 would suppress inflammation by regulating CCL-1 production. We explored the potential intracellular mechanisms, including epigenetic regulation on 4-PSB-2-suppressed CCL-1 production in human monocytes.

## 2. Materials and Methods

### 2.1. Cell Preparation

THP-1 human monocytic cell (American Type Culture Collection, Manassas, VA, USA) was cultured in RPMI 1640 medium, which was added with 10% fetal bovine serum and antibiotic-antimycotic (Gibco, Carlsbad, CA, USA) in a humidified incubator. THP-1 cells were cultured with 20 ng/mL phorbol 12-myristate 13-acetate (PMA; Sigma-Aldrich, Saint Louis, MO, USA) for 24 h to differentiate them into macrophages. The study protocol was authorized by the Institution Review Board of Kaohsiung Medical University Hospital (KMUHIRB-E(I)-20170005). Peripheral blood samples were acquired from healthy people who do not have a personal or family history of allergic disease (*n* = 3) after signing permits. Human primary CD14^+^ cells were isolated from peripheral blood mononuclear cells by magnetic bead sorting with anti-CD14 monoclonal antibody (Miltenyi Biotec, Bergisch Gladbach, NRW, Germany). Purity of isolated CD14^+^ cells used in the experiments was >95%.

### 2.2. Cell Viability

Various concentrations of 4-PSB-2 treatment were incubated in 96-well plates for 24 or 48 h. WST-1 (Sigma-Aldrich) activation solution was added and diluted at a ratio of 1:10. After the reaction solution was added, the plate was incubated for 1 h. The cell viability was analyzed via calculating the absorbance of the sample with an enzyme-linked immunosorbent assay (ELISA) reader (Bio-Rad Benchmark Plus microplate spectrophotometer, Bio-Rad Laboratories, Hercules, CA, USA) at a wavelength of 450 nm (the reference wavelength is 600 nm). The mean value was utilized to evaluate the cell viability and stated as a percentage of control.

### 2.3. Enzyme-Linked Immunosorbent Assay (ELISA)

The cells were pretreated with 4-PSB-2 for two hours or histone acetyltransferase inhibitor anacardic acid (AA) for 0.5 h before LPS (0.2 µg/mL) (Escherichia coli; Sigma-Aldrich). After being stimulated by LPS, the cell supernatant was harvested at 24 h and 48 h. The CCL-1 concentration was calculated by commercially available ELISA-based assay systems (R&D System, Minneapolis, MN, USA). ELISA assays were performed with recommended protocols by the manufacturer.

### 2.4. Western Blotting

After treating for 2 h with or without 4-PSB-2, the cells were stimulated with LPS then lysed with equal volumes with lysis buffer. Then, an equal amount of cell lysates was blotted and analyzed by Western blot with anti-p65, anti-phospho-p65, anti-mitogen-activated protein kinase (MAPK) (p38, ERK, and JNK), and anti-phospho-MAPK (p-p38, p-ERK, and p-JNK) antibodies (Cell Signaling Technology, Danvers, MA, USA). Immunoreactive bands were visualized by using horseradish-peroxidase-conjugated secondary antibody and the enhanced chemiluminescence (ECL) system (Merck Millipore, Burlington, MA, USA). The assay was performed by using the protocols recommended by the manufacturer.

### 2.5. Chromatin Immunoprecipitation (ChIP)

ChIP assays were conducted as in our previous research [16]. THP-1 cells were treated with 4-PSB-2 (10 and 20 μM) for 2 h at first and then were stimulated with LPS for 30 min. Following this, the cells were collected and added with 1% formaldehyde for crosslinking protein to DNA. The cells were left at room temperature for 10 min and we then used mechanical sonication to lyse them with chromatin fragmentation. After sonication, immunoprecipitation was performed overnight at 4 °C with anti-acetyl histone H3 or H4 antibodies (Abcam, Cambridge, UK). The above immune complexes used quantitative real-time polymerase chain reaction (qRT-PCR) to quantify the amplified product relative amounts by specific primers for promoter regions of *CCL1* genes and normalized them to the total input DNAs. The primers (sense: 5′-TTGCCTGTGCTGGTCTGACT; anti-sense: 5′-GTGGGATTGCTGGGTCAAAT) were designed, encompassing the following subregions relative to the transcription start sites: *CCL1* (−2964/−2661, containing NF-κB binding site) [17].

### 2.6. Statistical Analyses

The densitometric data from Western blot analysis were analyzed by ImageJ software (National Institutes of Health, Bethesda, MD, USA). All data were evaluated by Graphpad Prism 5 software (GraphPad Software Inc., Version 5.01, La Jolla, CA, USA), and the results were presented as mean ± standard deviation (SD). One-way ANOVA was utilized to analyze the differences between the experiment with the control groups. A *p*-value < 0.05 was considered as an indication of significant differences.

## 3. Results

### 3.1. 4-PSB-2 Suppressed LPS-Induced CCL-1 Production in THP-1 Cells

**Figure 1:**
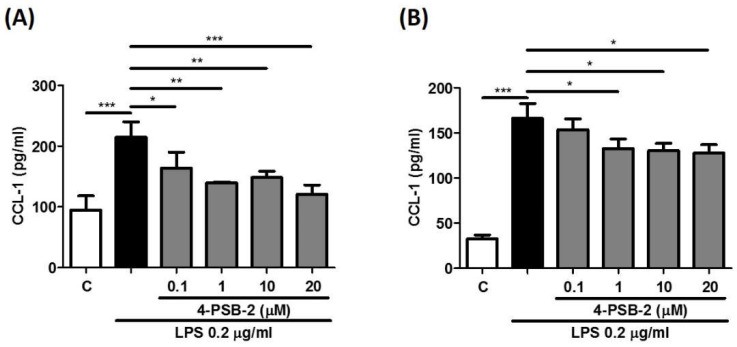
4-PSB-2 was able to suppress the LPS-induced CCL-1 production in THP-1 cells. After pretreatment with medium alone (**C**, white bar) or 4-PSB-2 (0.1, 1, 10, and 20 μM) for 2 h followed by LPS stimulation, the CCL-1 concentration was determined for 24 h (**A**) and 48 h (**B**). Data present the means ± SD of 3 independent experiments. (* *p* < 0.05, ** *p* < 0.01, and *** *p* < 0.001).

In the human immune system, monocytes and their derived cells are the major producers of cytokine and chemokine production in response to the antigen challenge [18,19,20,21]. To confirm our hypothesis, we explored the effect of 4-PSB-2 on CCL-1 expression in THP-1 cells. 4-PSB-2 significantly attenuated LPS-induced CCL-1 production in THP-1 cells at 24 and 48 h after LPS stimulation (from 0.1 to 20 µM) (Figure 1A,B).

### 3.2. The Experimental Concentration of 4-PSB-2 Had No Cytotoxic Effects on THP-1 Cells

**Figure 2:**
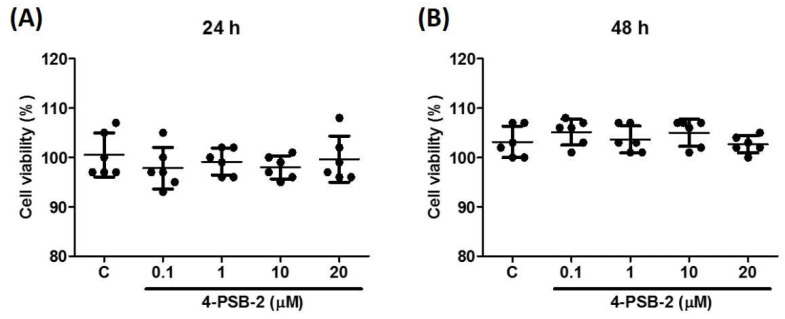
4-PSB-2 had no cytotoxic effect in THP-1 cells. After treatment with medium alone (**C**) or 4-PSB-2, the cell viability of THP-1 cells was measured for 24 h (**A**) and 48 h (**B**). Data present the means ± SD of 3 independent experiments.

We further investigated whether this suppressive effect of 4-PSB-2 was affected by cytotoxicity. To evaluate the effect of 4-PSB-2 on viability in THP-1 cells, we used WST-1 assay to determine the cytotoxic effect of 4-PSB-2 on THP-1 cells (at concentrations from 0.1 to 20 µM). After incubating for 24 and 48 h, 4-PSB-2 showed no significant cell viability suppression in THP-1 cells compared with the control group (Figure 2A,B).

### 3.3. 4-PSB-2 Suppressed CCL-1 Production in THP-1-Cell-Derived Macrophage and Human CD14^+^ Cells

**Figure 3:**
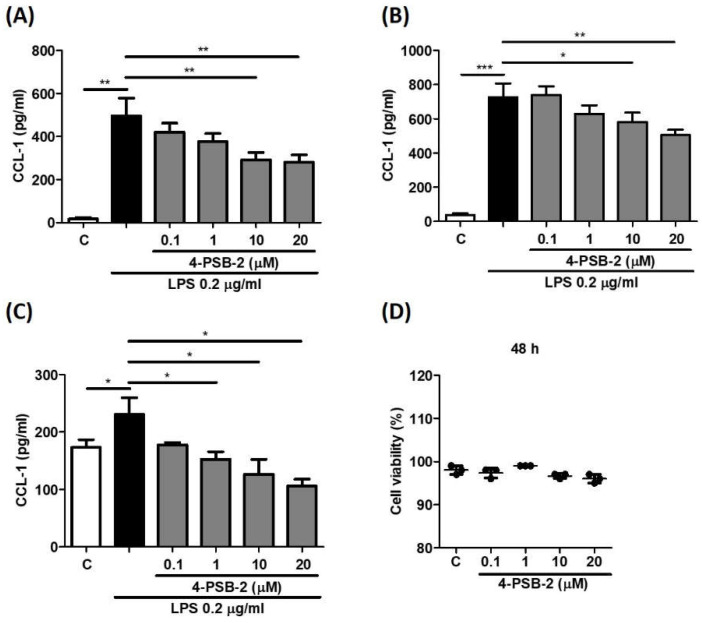
4-PSB-2 significantly decreased LPS-induced CCL-1 production in THP-1-derived macrophages and human primary CD14^+^ cells. After pretreatment with medium alone (**C**) or 4-PSB-2 (0.1, 1, 10, and 20 μM) for 2 h followed by LPS stimulation, the CCL-1 concentration was determined for 24 h (**A**) and 48 h (**B**) in THP-1-derived macrophages, or for 48 h (**C**) in human primary CD14^+^ cells. After treatment with medium alone (**C**) or 4-PSB-2, the cell viability of CD14^+^ cells was measured for 48 h (**D**). Data present the means ± SD of 3 independent experiments. (* *p* < 0.05, ** *p* < 0.01, and *** *p* < 0.001).

At both 24 h and 48 h after LPS stimulation, 4-PSB-2 was able to significantly reduce CCL-1 expression in THP-1-cell-derived macrophages at concentrations of 10 and 20 µM (Figure 3A,B). We also explored whether 4-PSB-2 had a similar influence on CCL-1 production in human CD14^+^ cells, which showed a significant reduction of CCL-1 production at 48 h after LPS stimulation (from 1 to 20 µM) (Figure 3C). As shown in Figure 3D, after being treated for 48 h, 4-PSB-2 showed no significant cell viability suppression in human CD14^+^ cells compared with the control group.

### 3.4. 4-PSB-2 Suppressed CCL-1 Expression through MAPK and NF-κB Pathways

**Figure 4:**
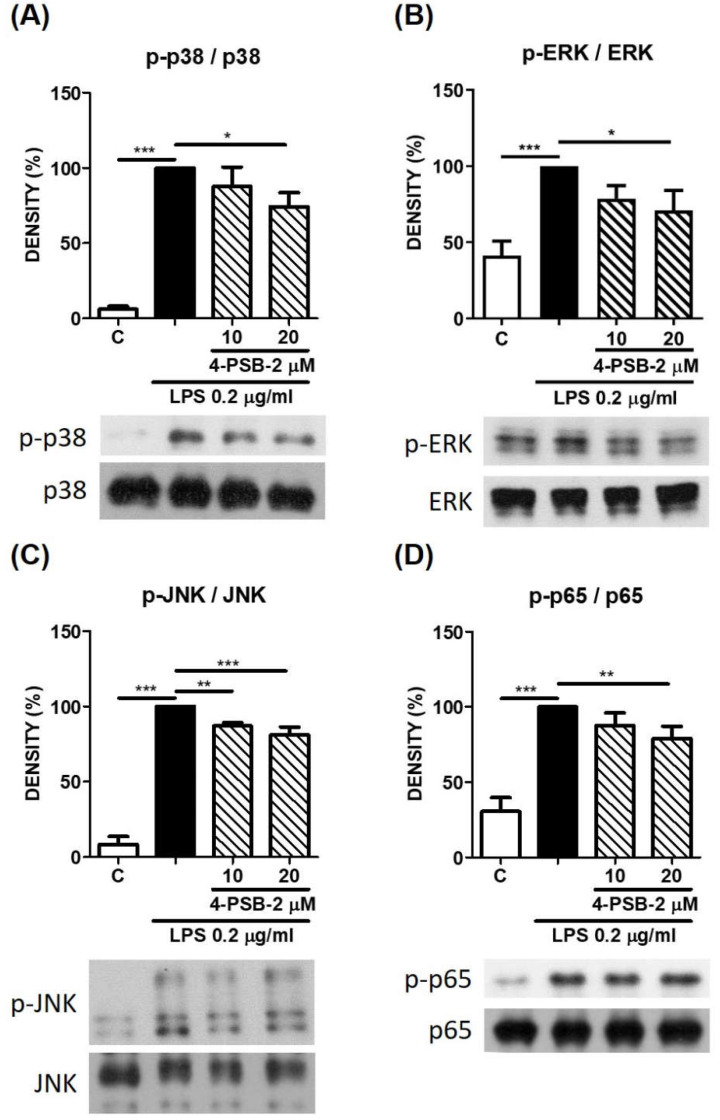
4-PSB-2 could significantly suppress LPS-induced p-MAPK and p-p65 expression in THP-1 cells. After pretreatment with medium alone (**C**) or 4-PSB-2 for 2 h followed by LPS stimulation for 1 h, the p-p38 (**A**), p-ERK (**B**), p-JNK (**C**), and p-p65 (**D**) expression were determined by Western blot. Data present the means ± SD of 3 independent experiments. (* *p* < 0.05, ** *p* < 0.01, and *** *p* < 0.001).

The MAPK pathway involves multiple inflammatory reactions, and LPS is considered as an activator for NF-κB and several MAPK pathways in human monocytes [18]. In previous research, it has been found that LPS-induced Th2-related cytokine CCL-22 expression depends on the MAPK-p38/JNK pathways without involving the MAPK-ERK pathway [22]. Therefore, we next examined whether 4-PSB-2 suppressed CCL-1 production through the MAPK pathway. As shown in Figure 4, 4-PSB-2 could suppress LPS-induced phosphorylation of p38, ERK, JNK, and p65 (Figure 4A–D). These results suggested that 4-PSB-2 may inhibit LPS-induced CCL-1 production by MAPK and NF-κB pathways.

### 3.5. 4-PSB-2 Suppressed LPS-Induced CCL-1 via Histone Acetylation

**Figure 5:**
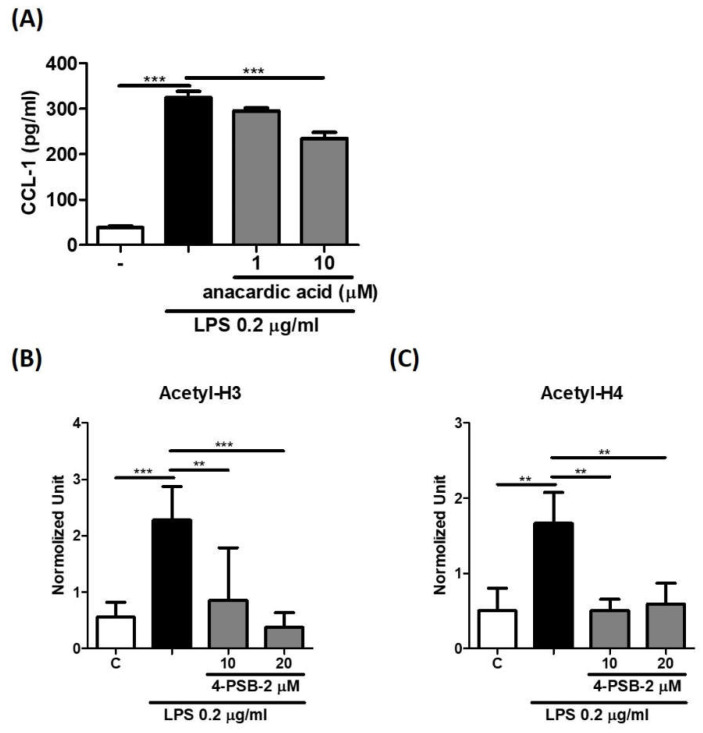
4-PSB-2 significantly downregulated LPS-induced CCL1 expression in THP-1 cells via histone acetylation. After pretreatment with medium alone (**C**) or AA for 1 h followed by LPS stimulation for 24 h, the CCL-1 production (**A**) was measurement by ELISA. After pretreatment with medium alone (**C**) or 4-PSB-2 for 2 h followed by LPS stimulation for 0.5 h, the acetylation of histone H3 (**B**) and histone H4 (**C**) at the promoter regions of *CCL1* gene were assessed by ChIP. Data present the means ± SD of 3 independent experiments. (** *p* < 0.01, and *** *p* < 0.001.)

Epigenetic regulations including histone modifications play critical roles in gene expression [23]. We used histone acetyltransferase inhibitor anacardic acid (AA) to evaluate whether histone acetylation was related to the suppression effect of 4-PSB-2 in CCL-1 production. The level of LPS-induced CCL-1 expression was decreased by AA (Figure 5A). In addition, ChIP assay results showed that 4-PSB-2 could suppress LPS-induced histone H3 and H4 acetylation at the proximal promoter subregion of the *CCL1* gene in THP-1 cells (Figure 5B,C). The above results revealed that 4-PSB-2 may suppress CCL-1 production via downregulating histone H3 and H4 acetylation at the *CCL1* gene region.

## 4. Discussion

In the past several years, natural products extracted from soft corals have been shown to be effective in treating inflammation disorders in rats [1,24,25]. The bioactivity of these natural products includes anti-inflammatory and neuroprotective activities [25,26]. Dihydroaustrasulfone alcohol, a synthetic precursor of austrasulfone, had been found with multiple bioactivities including anti-inflammation, neuroprotection, anti-tumorigenic, and anti-atherogenic abilities [7,8,9,27]. In addition, previous research proved that 4-PSB-2 had anti-inflammatory and neuroprotective properties by suppressing the expression of pro-inflammatory proteins including TNF-α, COX-2, and iNOS in both rats’ optic nerve cells [7] and human retinal pigment epithelium cells [27]. The latest study found that 4-PSB-2 had a suppressive effect on the production of TNF-α, COX-2, and iNOS by regulating the NF-κB pathway in human retinal pigment epithelial cells. This finding implies that 4-PSB-2 could be a potential medication in managing age-related macular degeneration [9].

Chemokines take an important place in the pathogenesis of allergy response with the ability to recruit inflammatory cells, activate Th2 responses, and contribute to airway remodeling in asthma patients [11,12]. Thus, several chemokines may be the potential treatment target in the management of asthma [12,28]. CCL-1, a chemokine that can bind to CC chemokine receptor 8, is widely expressed on inflammatory cells including helper and cytotoxic T cells, eosinophils, and T regulatory cells [29]. In our research, we found that 4-PSB-2 suppressed CCL-1 expression in monocytes, which indicates the potential role of 4-PSB-2 in treating allergic diseases and asthma. CCL-1 also has a prominent place in inflammation and apoptosis, angiogenesis, and tumor biology [30]. Neutralizing of CCL-1 can be utilized as anti-tumor immunotherapy by suppressing the immunosuppressive activity of regulatory T cells [31]. Moreover, 4-PSB-2 could potently diminish the toll-like receptor (TLR)-related CCL-1 expression in THP-1 cells and primary monocytes. Therefore, this suppression effect of 4-PSB-2 on CCL-1 may further enhance the anti-tumor ability. In addition, we investigated the intracellular signal pathway to find out the precise underlying mechanism. As we know, MAPKs and NF-κB pathways are related to the regulation of LPS-induced chemokines with cytokines expression in monocytes [22]. Our previous publication also found that LPS-induced CCL-1 production was regulated through the MAPKs pathway [32]. Our work revealed that 4-PSB-2 may suppress the activation of p65, JNK, ERK, and p38 by LPS. Thus, MAPKs and NF-κB may have roles in these suppression effects of 4-PSB-2 on the CCL-1 production of LPS-stimulated monocytes.

Several studies have demonstrated that histone modification affects different aspects of allergic disease, including the effectiveness of pharmacological therapies. Histone acetylation such as H3 and H4 panacetylation, or methylation at specific sites increases gene transcription, which is manipulated by histone acetyltransferases or methyltransferase [15]. In previous research, we found that 2,3,7,8-tetrachlorodibenzo-p-dioxin (TCDD) could stimulate CCL-1 secretion in THP-1-derived M2 macrophages. In addition, TCDD-induced CCL-1 production was regulated by H3K27 tri-methylation at the distal dioxin-responsive element (DRE) in the CCL1 promoter region [33]. Glucocorticoids, a common anti-inflammation medication, have been indicated as being able to inhibit HAT activity, recruiting histone deacetylase-2 and leading to suppression of these activated inflammatory genes [34]. In this study, our data showed that acetyl-H3 and H4 levels were significantly upregulated in LPS-induced CCL-1 production, and 4-PSB-2 could suppress H3 and H4 histone acetylation at the CCL1 promoter region.

Our present study had a novel finding in that we discovered the thorough intracellular pathways including epigenetic modulation of this suppressive effect of 4-PSB-2, a marine natural product from soft coral, on the expression of Th2-related chemokine in monocytes with macrophages. It is well known that Th2-related chemokines including CCL-1 are strongly related to the pathogenesis of allergic diseases [11,12,28]. In this study, we first confirmed that 4-PSB-2 suppressed LPS-induced CCL-1 expression, and this result further indicated that 4-PSB-2 may have a potential role in treating or preventing allergic diseases. Next, we showed the effect of 4-PSB-2 on the expression of CCL-1 was regulated by the MAPKs and NF-κB pathway. Moreover, we also confirmed that 4-PSB-2 attenuated LPS-induced expression of CCL-1 via histone H3 and H4 acetylation at the CCL1 promoter region. As far as we know, this is the first research to provide evidence that marine natural products can suppress CCL-1 production in human monocytes via epigenetic regulation.

In our research, the marine natural product 4-PSB-2 was found to be an immune modulator to suppress Th2 chemokine CCL-1 expression in monocytes. Therefore, 4-PSB-2 may have anti-allergic effects via suppressing CCL-1 production of monocytes and macrophages. Further animal model experiments with human trials are necessary to clarify the clinical advantages of 4-PSB-2 in treating allergy diseases. Our research increases the knowledge of the possible therapeutic mechanisms in 4-PSB-2, which may be developed as an alternative strategy in protecting asthma and allergy diseases against progressive deterioration. 

## Data Availability

The data presented in this study are available on request from the corresponding author. Data may be available upon request to interested researchers. Please send data requests to Chih-Hsing Hung, MD, PhD. Department of Pediatrics, Kaohsiung Medical University Hospital, Kaohsiung Medical University.
